# Association between pathologic complete response and biochemical indicators after neoadjuvant therapy for HER2-positive breast cancer

**DOI:** 10.1186/s12957-024-03366-w

**Published:** 2024-05-09

**Authors:** Wei Chen, Jing Zhang, Fenxiang Li, Zongshun Chen, Junjie Li, Da-Lin Lu

**Affiliations:** 1 Breast Surgery Department, Sichuan Clinical Research Center for Cancer, Sichuan Cancer Hospital and Institute, Sichuan Cancer Center, Affiliated Cancer Hospital of University of Electronic Science and Technology of China, Chengdu, China No. 55, Section 4, Renmin South Road, Sichuan; 2 School of Medical and Life Sciences, Chengdu University of Traditional Chinese Medicine, Chengdu, China; 3 Department of Epidemiology, School of Medicine, Jinan University, Guangzhou, China No. 601, Huangpu Avenue West, Tianhe District 510632

**Keywords:** HER2-positive breast cancer, Neoadjuvant therapy, Biochemical markers, Predicting pathologic complete response, Glycemic and lipid management

## Abstract

**Purpose:**

This study investigated the changes in the fasting blood glucose (FBG), fasting triglyceride (FTG), and fasting total cholesterol (FTC) levels during neoadjuvant therapy (NAT) for human epidermal growth factor receptor 2 (HER2)-positive breast cancer (BC) and the association with pathologic complete response (pCR).

**Methods:**

Relevant data from Sichuan Cancer Hospital from June 2019 to June 2022 were collected and analyzed, and FBG, FTG, and FTC were divided into baseline, change, and process groups, which were grouped to analyze the changes after receiving NAT and the association with pCR.

**Results:**

In the estrogen receptor (ER)-negative subgroup, patients with low levels of FTG in the process group were more likely to achieve pCR compared to high levels, and in the progesterone receptor (PR)-negative subgroup, patients with lower FTG compared to higher FTG after receiving NAT was more likely to achieve pCR.

**Conclusions:**

Patients with HER2-positive BC undergoing NAT develop varying degrees of abnormalities (elevated or decreased) in FBG, FTG, and FTC; moreover, the status of FTG levels during NAT may predict pCR in ER-negative or PR-negative HER2-positive BC.Early monitoring and timely intervention for FTG abnormalities may enable this subset of patients to increase the likelihood of obtaining a pCR along with management of abnormal markers.

## Highlights

• Neoadjuvant therapy causes various fasting glucose, fasting triglyceride, and fasting cholesterol abnormalities.

• Fasting triglycerides may predict the pathologic complete response in some patients.

• Fasting blood glucose and fasting total cholesterol do not predict the pathologic complete response.

• Some patients may derive both short-term (achievement of pathologic complete response) and long-term (reduction of cardiovascular disease risk) benefits in conjunction with lipid management.

## Background

Breast cancer (BC) represents the most common malignant tumor in women, posing a severe health threat [1]. Furthermore, 15% ~ 20% of BC are human epidermal growth factor receptor 2 (HER2)-positive, which is clinically characterized by strong invasiveness and poor prognosis [2, 3]. Neoadjuvant therapy (NAT) is the standard preferred initial treatment for patients with HER2-positive BC (tumor primary stage ≥ 2 or lymph node status stage ≥ 1). This includes the TCbHP regimen of trastuzumab (H) and pertuzumab (P) in combination with paclitaxel and platinum drugs, and the A/EC-THP regimen of cyclophosphamide in combination with anthracycline sequenced with paclitaxel in combination with HP drugs [4, 5].

Pathologic complete response (pCR) is widely used for the alternative assessment of NAT efficacy since patients who achieve a pCR after receiving NAT have longer event-free survival (EFS) and overall survival (OS) periods [6–9]. Therefore, studies on clinicopathologic factors predicting pCR in HER2-positive BC are emerging, including hormone receptor (HR) expression status [10], histologic grading [11], and clinical staging [12].

However, receiving NAT to obtain pCR may cause or exacerbate biochemical marker abnormalities, including fasting blood glucose (FBG), fasting triglycerides (FTG), and fasting total cholesterol (FTC), increasing the risk of other diseases, especially cardiovascular disease (CVD) risk [13–17]. The association between these commonly used, highly reproducible, inexpensive, and convenient biochemical markers and NAT and pCR in HER2-positive BC has not yet been established.

This study analyzes the FBG, FTG, and FTC association with the existing NAT regimen and pCR to provide medical evidence for early HER2-positive BC glycemic, lipid management and pCR prediction.

## Methods

### Subjects and research design

**Fig. 1:**
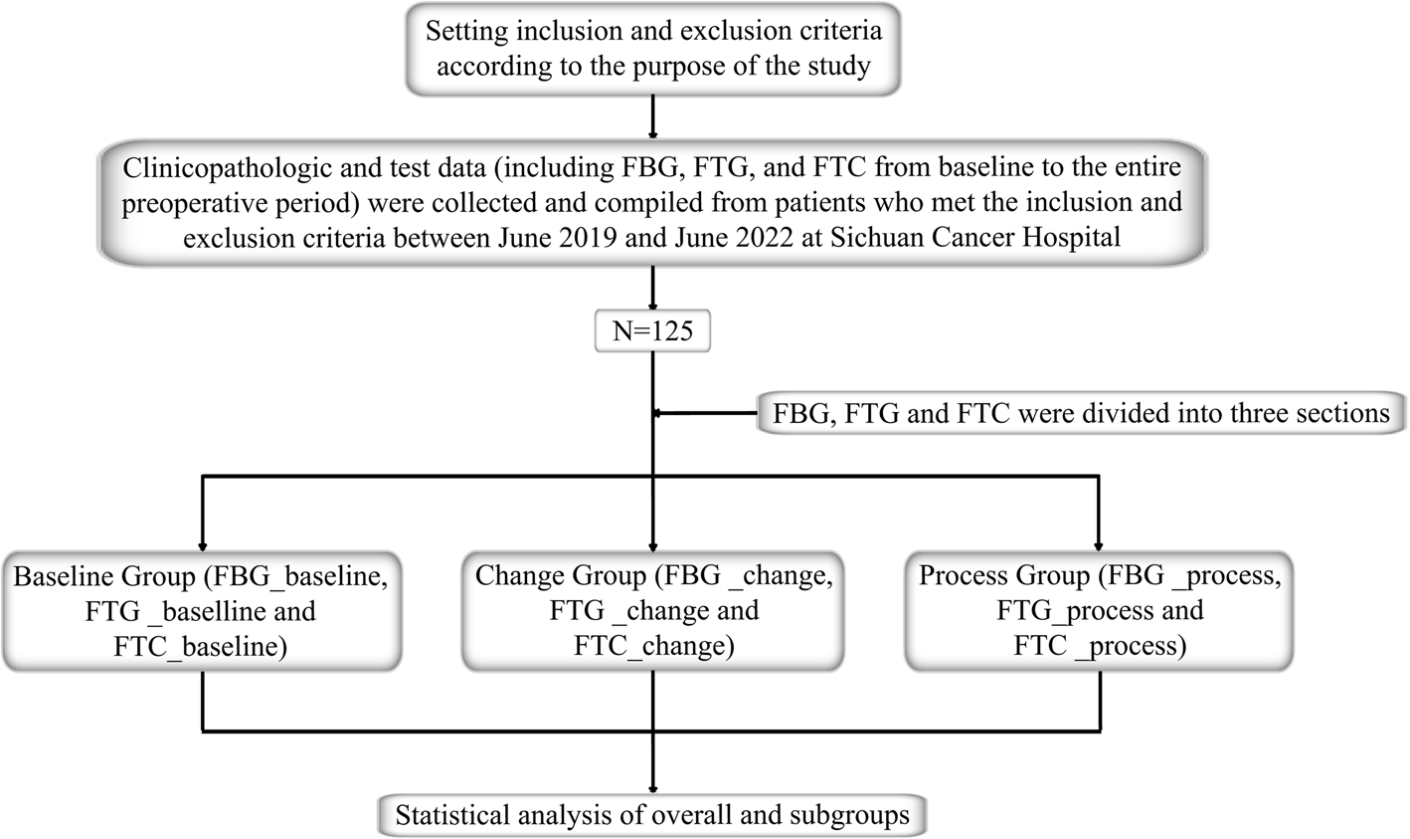
Technological route

This study examined the clinical and pathologic data of HER2-positive invasive BC patients who were first diagnosed and underwent surgery after combined neoadjuvant chemotherapy (NAC) and dual-targeted therapy (regimen: TCbHP or A/EC-THP) at the Breast Surgery Center of Sichuan Cancer Hospital from June 2019 to June 2022 (Fig. 1).

### Inclusion and exclusion criteria

The study included (1) females, (2) first diagnosed with HER2-positive invasive BC at the hospital, (3) clinical stages II or III, (4) unilateral BC, (5) complete and available baseline-to-preoperative FBG, FTG, and FTC profiles in fasting venous blood, (6) complete clinical and pathologic data, and (7) those receiving TCbHP or A/EC-THP as the NAT treatment regimen.

The study excluded (1) males, (2) surgery without standardized NAT combined with dual-targeted therapy, (3) incomplete test, clinical, or pathologic data, (4) stages I or IV BC, (5) patients receiving weekly treatments, (6) those with previously diagnosed cancers at the time of admission, (7) occult BC, (8) those who changed their regimen during the NAT period, (9) those who did not undergo the full treatment course at the hospital, and (10) FBG, FTG, and FTC test data from non-fasting venous blood.

### Pathologic interpretation criteria

Estrogen receptor (ER) and progesterone receptor (PR) status were determined by immunohistochemistry (IHC), with ER and PR positivity [18] defined as positive nuclear staining in at least 1% of tumor cells, and HR negativity defined as negative for both ER and PR; otherwise, it was considered HR positivity. HER2 status [19] was determined by IHC and fluorescence in-situ hybridization (FISH), with HER2 3 + and HER2 2 + /FISH-positivity for HER2 positivity.

### Clinical interpretation criteria

The clinical staging was guided by the eighth edition of the American Joint Committee on Cancer [20]. The menstrual status was interpreted in conjunction with questioning at the time of first diagnosis, age, and laboratory tests [21]. The reference ranges for follicle-stimulating hormone and estradiol in our hospital were (follicular phase 3.5–12.5, ovulatory phase 4.7–21.5, luteal phase 1.7–7.7, and menopausal phase 25.8–134.8) mIU/mL and (follicular phase 12.4–233, ovulatory phase 41–398, luteal phase 22.3–341, and menopausal phase < 5–138) pg/ml, respectively.

### Biochemical interpretation criteria

**Fig. 2:**
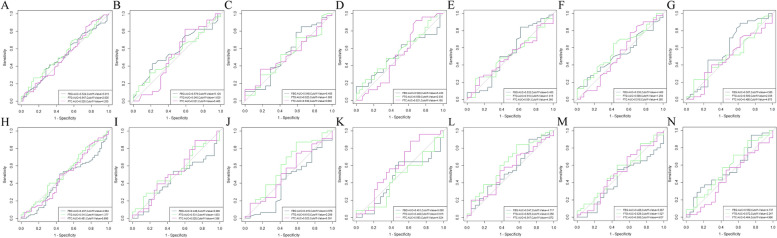
The overall and subgroup ROC curves: ROC curves of FBG, FTG, and FTC for the baseline and process groups in the overall analysis (with information on the cutoff value and AUCs) presented in Figs A and H, respectively; and ROC curves of FBG, FTG, and FTC for the baseline and process groups in the analysis of the ER-positive and ER-negative subgroups (with information on the cutoff value and AUCs) presented in Figs B, I, and C, and J, respectively.ROC curves of FBG, FTG, and FTC for baseline and process groups in PR-positive and PR-negative subgroup analyses (with information on cutoff values and AUCs) presented in Figs D, K, and E, and L, respectively; and ROC curves of FBG, FTG, and FTC for baseline and process groups in menopausal and non-menopausal subgroup analyses presented in Figs F, M, and G, and N, respectively (with information on information on cutoff values and AUCs)

The baseline patient group information was obtained from the test data at the first visit to the hospital (corresponding to the period before the start of the first NAT). The process group data was obtained from the average values of the period before the second NAT to the preoperative period. The FBG, FTG, and FTC reference ranges in the hospital were 3.89–6.11 mmol/L, 0.00–1.70 mmol/L, and 0.00–5.20 mmol/L, respectively, which might be interpreted differently by different testing instruments. Criteria for high and low FBG, FTG, and FTC interpretation in the baseline and process groups were determined by the cutoff values of the receiver operating characteristic (ROC) curves, whereas high and low FBG, FTG, and FTC interpretation in the change group was determined by comparing the sizes of the process and baseline groups (Fig. 2).

### Efficacy evaluation

Since pCR is widely used as an alternative prognostic indicator for EFS and OS [6–9], this study assessed the efficacy of combined NAC and dual-targeted therapy in terms of whether pCR (ypT0/isypN0) was achieved [22].

### Statistical methodology

Wilcoxon's rank-sum test was used to analyze the changes in the levels of FBG, FTG and FTC after NAC combined with dual-targeted therapy (*p* < 0.05 indicated statistically significant differences). The ROC curves were plotted using the R language (R 4.3.1) to determine the FBG, FTG, and FTC cutoff values in the groups, showing a change from continuous to dichotomous variables. Univariate and multivariate logistic regression analyses were performed using SAS 9.4 to examine the FBG, FTG, and FTC association with the pCR in the different groups (*p* < 0.05 indicated statistically significant differences).

## Results

### NAT affects FBG, FTG, and FTC

Combining NAC with dual-targeted therapy increased the FBG levels in both the overall and subgroup analyses, yielding statistically significant differences [(5.07 ± 1.19) mmol/L vs. (5.19 ± 0.76) mmol/L, (5.01 ± 0.86) mmol/L vs. (5.15 ± 0.70) mmol/L, (5.14 ± 1.44) mmol/L vs. (5.23 ± 0.82) mmol/L, (5.22 ± 1.41) mmol/L vs. (5.34 ± 0.90) mmol/L, (4.91 ± 0.85) mmol/L vs. (5.02 ± 0.54) mmol/L, (4.91 ± 0.86) mmol/L vs. (5.07 ± 0.55) mmol/L, and (5.20 ± 1.38) mmol/L vs. (5.28 ± 0.89) mmol/L, all *p* < 0.05] (Table 1).

**Table 1:** Comparison of differences between baseline and process groups

	Baseline group (mean ± std)	Process group (mean ± std)	*P*-value
**Total(*n* = 125)**			
FBG	5.07 ± 1.19	5.19 ± 0.76	** < 0.001**
FTG	1.59 ± 0.93	1.79 ± 0.85	** < 0.001**
FTC	5.04 ± 0.90	5.00 ± 0.72	0.791
**ER + (*n* = 61)**			
FBG	5.01 ± 0.86	5.15 ± 0.70	**0.020**
FTG	1.53 ± 0.98	1.74 ± 0.81	** < 0.001**
FTC	4.94 ± 0.90	4.97 ± 0.68	0.370
**ER-(*n* = 64)**			
FBG	5.14 ± 1.44	5.23 ± 0.82	** < 0.001**
FTG	1.64 ± 0.89	1.85 ± 0.89	** < 0.001**
FTC	5.14 ± 0.90	5.03 ± 0.75	0.192
**Menopause(*n* = 66)**			
FBG	5.22 ± 1.41	5.34 ± 0.90	** < 0.001**
FTG	1.80 ± 1.06	1.99 ± 0.95	**0.002**
FTC	5.30 ± 0.88	5.06 ± 0.69	**0.013**
**Non-menopause(*n* = 59)**			
FBG	4.91 ± 0.85	5.02 ± 0.54	**0.013**
FTG	1.34 ± 0.70	1.58 ± 0.68	** < 0.001**
FTC	4.76 ± 0.84	4.93 ± 0.75	**0.010**
**PR + (*n* = 54)**			
FBG	4.91 ± 0.86	5.07 ± 0.55	**0.003**
FTG	1.51 ± 1.02	1.67 ± 0.78	**0.004**
FTC	4.960 ± 0.91	4.961 ± 0.63	0.640
**PR-(*n* = 71)**			
FBG	5.20 ± 1.38	5.28 ± 0.89	**0.002**
FTG	1.64 ± 0.86	1.88 ± 0.90	** < 0.001**
FTC	5.11 ± 0.89	5.03 ± 0.78	0.308

Reference range: FBG (3.89–6.11 mmol/L), FTG (0.00–1.70 mmol/L), and FTC (0.00–5.20 mmol/L)

Combining NAC with dual-targeted therapy increased the FTG levels in both the overall and subgroup analyses, yielding statistically significant differences [(1.59 ± 0.93) mmol/L vs. (1.79 ± 0.85) mmol/L, (1.53 ± 0.98) mmol/L vs. (1.74 ± 0.81) mmol/L, (1.64 ± 0.89) mmol/L vs. (1.85 ± 0.89) mmol/L, (1.80 ± 1.06) mmol/L vs. (1.99 ± 0.95) mmol/L, (1.34 ± 0.70) mmol/L vs. (1.58 ± 0.68) mmol/L, (1.51 ± 1.02) mmol/L vs. (1.67 ± 0.78) mmol/L, and (1.64 ± 0.86) mmol/L vs. (1.88 ± 0.90) mmol/L, all *p* < 0.05] (Table 1).

Combining NAC combined with dual-targeted therapy reduced the FTC levels in the overall, menopausal, ER-negative, and PR-negative subgroups [(5.04 ± 0.90) mmol/L vs. (5.00 ± 0.72) mmol/L, (5.30 ± 0.88) mmol/L vs. (5.06 ± 0.69) mmol/L, (5.14 ± 0.90) mmol/L vs. (5.03 ± 0.75) mmol/L, and (5.11 ± 0.89) mmol/L vs. (5.03 ± 0.78)) mmol/L], and increased these levels in the non-menopausal, ER-positive, and PR-positive subgroups [(4.76 ± 0.84) mmol/L vs. (4.93 ± 0.75) mmol/L, (4.94 ± 0.90) mmol/L, ( 4.94 ± 0.90) mmol/L vs. (4.97 ± 0.68) mmol/L, and (4.960 ± 0.91) mmol/L vs. (4.961 ± 0.63) mmol/L]. The differences were only statistically significant between the menopausal and non-menopausal subgroups (*p* < 0.05) (Table 1) (Fig. 2).

### The overall baseline characterization and analysis

This study included HER2-positive patients (*N* = 125) meeting the enrollment criteria. Univariate and multivariate statistical analyses showed that the overall sample pCR rate was approximately 60.00%, while no other independent influencing factors predicted pCR, except for the HER2 expression status (pCR: 35.00% vs. 64.76%, OR = 3.413, 95% CI: 1.253–9.299, *P* = 0.0164) (Table 2).

**Table 2:** Analysis of patient characteristics on pCR (*N* = 125)

Variable	non-pCR N(%)	pCR N(%)	Univariate analysis *P*-value	Multivariate analysis ^a^	
				OR(95%CI)	*P*-value
**Total (N)**	50(40.00)	75(60.00)			
**Age**			0.2210		
≤ 35	8(57.14)	6(42.86)			
35 < age ≤ 55	31(41.33)	44(58.67)			
> 55	11(30.56)	25(69.44)			
**Menstrual status**			0.1090		
Non-menopause	28(47.46)	31(52.54)			
Menopause	22(33.33)	44(66.67)			
**Clinical stage**			0.1375		
II stage	26(34.67)	49(65.33)			
III stage	24(48.00)	26(52.00)			
**HR status**			**0.0349**		0.1009
Negative	17(29.82)	40(70.18)		Ref	
Positive	33(48.53)	35(51.47)		0.528(0.246–1.133)	
**HER2 expression state**			**0.0164**		**0.0164**
2 + /FISH +	13(65.00)	7(35.00)		**Ref**	
3 +	37(35.24)	68(64.76)		**3.413(1.253–9.299)**	
**Neoadjuvant therapy**			0.4076		
A/EC-THP	21(44.68)	26(55.32)			
TCbHP	29(37.18)	49(62.82)			
**FBG_baseline**^**b**^			0.2924		
Low	34(43.59)	44(56.41)			
High	16(34.04)	31(65.96)			
**FTG_baseline**^**c**^			0.0968		
Low	43(43.88)	55(56.12)			
High	7(25.93)	20(74.07)			
**FTC_baseline**^**d**^			0.1191		
Low	13(54.17)	11(45.83)			
High	37(36.63)	64(63.37)			
**FBG_process**^**e**^			0.4652		
Low	22(36.67)	38(63.33)			
High	28(43.08)	37(56.92)			
**FTG_process**^**f**^			0.3644		
Low	16(34.78)	30(65.22)			
High	34(43.04)	45(56.96)			
**FTC_process**^**g**^			0.3980		
Low	39(38.24)	63(61.76)			
High	11(47.83)	12(52.17)			
**FBG_change**			0.8186		
Low	17(38.64)	27(61.36)			
High	33(40.74)	48(59.26)			
**FTG_change**			0.2248		
Low	11(31.43)	24(68.57)			
High	39(43.33)	51(56.67)			
**FTC_change**			0.5113		
Low	23(37.10)	39(62.90)			
High	27(42.86)	36(57.14)			

Abbreviations: *OR* Odds ratio, *CI* Confidence interval, *pCR* pathologic complete response, *non-pCR* non-pathologic complete response

A/EC-THP: *A/E* Anthracycline, *C* Cyclophosphamide, *T* Paclitaxel drugs, *H* Trastuzumab, *P* Pertuzumab. TCbHP: * T* Paclitaxel drugs, *Cb* Platinum drugs, *H* Trastuzumab, *P* Pertuzumab

^a^Logistic regression was used for analysis. All statistical tests were two-sided. The OR was calculated using the non-pCR as a reference. Adjusted for HR status and HER2 expression state

^b,c,d,e,f,g^ ROC curves refer to Fig. 2 [the best cutoff value for the baseline FBG value in the overall analysis conditions were 5.085 and 5.015, respectively, both of which had the same effect on the results, while 5.015 was ultimately selected as optimal]

### The subgroup analysis based on the ER status

The ER-negative subgroup analysis showed that the FTG level in the process group (pCR: 80.39% vs. 46.15%, OR = 0.209, 95% CI: 0.057–0.760, *P* = 0.0175) was an independent influencing factor of pCR. The ER-positive subgroup analysis indicated that the PR expression status (pCR: 71.43% vs. 38.80%, OR = 0.248, 95% CI: 0.068–0.911, *P* = 0.0357) was an independent predictor of pCR (Table 3).

**Table 3:** Analysis of the effect of the patient characteristics on pCR (ER-/ER +)

Variable	ER-(*n* = 64)					ER + (*n* = 61)				
	non-pCR N(%)	pCR N(%)	Univariate analysis *P*-value	Multivariate analysis^a^		non-pCR N(%)	pCR N(%)	Univariate analysis *P*-value	Multivariate analysis^b^	
				OR(95%CI)	*P*-value				OR(95%CI)	*P*-value
**Total (N)**	17(26.52)	47(73.48)				33(54.10)	28(45.90)			
**Age**			0.2835					0.9986		
≤ 35	2(66.67)	1(33.33)				6(54.55)	5(45.45)			
35 < age ≤ 55	10(27.78)	26(72.22)				21(53.85)	18(46.15)			
> 55	5(20.00)	20(80.00)				6(54.55)	5(45.45)			
**Menstrual status**			0.0652					0.5933		
Non-menopause	9(40.91)	13(59.09)				19(51.35)	18(48.65)			
Menopause	8(19.05)	34(80.95)				14(58.33)	10(41.67)			
**Clinical stage**			**0.0327**		0.1053			0.7677		
II stage	6(16.22)	31(83.78)		Ref		20(52.63)	18(47.37)			
III stage	11(40.74)	16(59.26)		0.365 (0.107–1.236)		13(56.52)	10(43.48)			
**PR expression**			0.9690					**0.0357**		**0.0357**
Negative	17(29.82)	40(70.18)				4(28.57)	10(71.43)		**Ref**	
Positive	0	7(100.00)				29(61.70)	18(38.80)		**0.248** **(0.068–0.911)**	
**HER2 expression state**			0.6944					**0.0456**		0.1308
2 + /FISH +	2(33.33)	4(66.67)				11(78.57)	3(21.43)		Ref	
3 +	15(25.86)	43(74.14)				22(46.81)	25(53.19)		3.056 (0.718–13.011)	
**Neoadjuvant therapy**			0.2032					0.7841		
A/EC-THP	8(36.36)	14(63.64)				13(52.00)	12(48.00)			
TCbHP	9(21.43)	33(78.57)				20(55.56)	16(44.44)			
**FBG_baseline**^**c**^			0.0813					0.0733		
Low	11(21.57)	40(78.43)				26(61.90)	16(38.10)			
High	6(46.15)	7(53.85)				7(36.84)	12(63.16)			
**FTG_baseline**^**d**^			0.3060					0.1678		
Low	7(35.00)	13(65.00)				31(57.41)	23(42.59)			
High	10(22.73)	34(77.27)				2(28.57)	5(71.43)			
**FTC_baseline**^**e**^			0.1677					0.0719		
Low	14(31.82)	30(68.18)				20(46.51)	23(53.49)			
High	3(15.00)	17(85.00)				13(72.22)	5(27.78)			
**FBG_process**^**f**^			0.7773					0.5279		
Low	8(25.00)	24(75.00)				15(50.00)	15(50.00)			
High	9(28.13)	23(71.88)				18(58.06)	13(41.94)			
**FTG_process**^**g**^			**0.0175**		**0.0175**			0.9748		
Low	10(19.61)	41(80.39)		**Ref**		0	3(100.00)			
High	7(53.85)	6(46.15)		**0.209** **(0.057–0.760)**		33(56.90)	25(43.10)			
**FTC_process**^**h**^			0.2308			9(75.00)	3(25.00)	0.1162		
Low	10(22.22)	35(77.78)				24(48.98)	25(51.02)			
High	7(36.84)	12(63.16)								
**FBG_change**			0.3060			10(41.67)	14(58.33)	0.1196		
Low	7(35.00)	13(65.00)				23(62.16)	14(37.84)			
High	10(22.73)	34(77.27)								
**FTG_change**			0.9522					0.6892		
Low	0	16(100.00)				11(57.89)	8(42.11)			
High	17(35.42)	31(64.58)				22(52.38)	20(47.62)			
**FTC_change**			0.2977					0.4417		
Low	8(21.62)	29(78.38)				15(60.00)	10(40.00)			
High	9(33.33)	18(66.67)				18(50.00)	18(50.00)			

Abbreviations: *OR* Odds ratio, *CI* Confidence interval, *pCR* pathologic complete response, *non-pCR* non-pathologic complete response

A/EC-THP: *A/E* Anthracycline, *C* Cyclophosphamide, *T* Paclitaxel drugs, *H* Ttrastuzumab, *P* Pertuzumab. TCbHP: *T*, Paclitaxel drugs, *Cb* Platinum drugs, *H* Trastuzumab, *P* Pertuzumab

^a^Logistic regression was used for analysis. All statistical tests were two-sided. The OR was calculated using the non-pCR as a reference. Adjusted for the clinical stage, FTG_process

^b^Logistic regression was used for analysis. All statistical tests were two-sided. The OR was calculated using the non-pCR as a reference. Adjusted for PR expression, HER2 expression state

^c,d,e,f,g,h^ ROC curves refer to Fig. 2

### The subgroup analysis based on the PR status

The PR-negative subgroup analysis showed that the clinical stage (pCR: 82.50% vs. 54.84%, OR = 0.234, 95% CI: 0.075–0.731, *P* = 0.0124) and triglyceride trend (pCR: 94.12% vs. 62.96%, OR = 0.095, 95% CI: 0.011–0.805, *P* = 0.0309) independently influenced pCR. No factors independently influencing pCR were present in the PR-positive subgroup (Table 4).

**Table 4:** Analysis of the effect of patient characteristics on pCR (PR-/PR +)

Variable	PR-(*n* = 71)					PR + (*n* = 54)				
	non-pCR N(%)	pCR N(%)	Univariate analysis *P*-value	Multivariate analysis^a^		non-pCR N(%)	pCR N(%)	Univariate analysis *P*-value	Multivariate analysis	
				OR(95%CI)	*P*-value				OR(95%CI)	*P*-value
**Total (N)**	21(29.58)	50(70.42)				29(53.70)	25(46.30)			
**Age**			0.4207					0.6256		
≤ 35	2(66.67)	1(33.33)				6(54.55)	5(45.45)			
35 < age ≤ 55	12(28.57)	30(71.43)				19(57.58)	14(42.42)			
> 55	7(26.92)	19(73.08)				4(40.00)	6(60.00)			
**Menstrual status**			0.4806					0.4752		
Non-menopause	9(34.62)	17(65.38)				19(57.58)	14(42.42)			
Menopause	12(26.67)	33(73.33)				10(47.62)	11(52.38)			
**Clinical stage**			**0.0138**		**0.0124**			0.9703		
II stage	7(17.50)	33(82.50)		**Ref**		19(54.29)	16(45.71)			
III stage	14(45.16)	17(54.84)		**0.234** **(0.075–0.731)**		10(52.63)	9(47.37)			
**ER expression**			0.9267					0.9609		
Negative	17(29.82)	40(70.18)				0	7(100.00)			
Positive	4(28.57)	10(71.43)				29(61.70)	18(38.30)			
**HER2 expression**			0.3712					0.1558		
2 + /FISH +	2(50.00)	2(50.00)				11(68.75)	5(31.25)			
3 +	19(28.36)	48(71.64)				18(47.37)	20(52.63)			
**Neoadjuvant therapy**			0.2254					0.6257		
A/EC-THP	9(39.13)	14(60.87)				12(50.00)	12(50.00)			
TCbHP	12(25.00)	36(75.00)				17(56.67)	13(43.33)			
**FBG_baseline**^**b**^			0.9295					0.9708		
Low	12(30.00)	28(70.00)				29(58.00)	21(42.00)			
High	9(29.03)	22(70.97)				0	4(100.00)			
**FTG_baseline**^**c**^			0.1770					0.1904		
Low	2(14.29)	12(85.71)				26(57.78)	19(42.22)			
High	19(33.33)	38(66.67)				3(33.33)	6(66.67)			
**FTC_baseline**^**d**^			0.2881					0.0502		
Low	3(18.75)	13(81.25)				9(81.82)	2(18.18)			
High	18(32.73)	37(67.27)				20(46.51)	23(53.49)			
**FBG_process**^**e**^			0.0577					0.2485		
Low	6(54.55)	5(45.45)				14(46.67)	16(53.33)			
High	15(25.00)	45(75.00)				15(62.50)	9(37.50)			
**FTG_process**^**f**^			**0.0193**		0.1007			0.9795		
Low	12(22.22)	42(77.78)		Ref		0	2(100.00)			
High	9(52.94)	8(47.06)		0.349 (0.099–1.227)		29(55.77)	23(44.23)			
**FTC_process**^**g**^			0.2244					0.0802		
Low	8(22.86)	27(77.14)				11(73.33)	4(26.67)			
High	13(36.11)	23(63.89)				18(46.15)	21(53.85)			
**FBG_change**			0.3837					0.2110		
Low	9(36.00)	16(64.00)				8(42.11)	11(57.89)			
High	12(26.09)	34(73.91)				21(60.00)	14(40.00)			
**FTG_change**			**0.0359**		**0.0309**			0.8473		
Low	1(5.88)	16(94.12)		**Ref**		10(55.56)	8(44.44)			
High	20(37.04)	34(62.96)		**0.095** **(0.011–0.805)**		19(52.78)	17(47.22)			
**FTC_change**			0.7798					0.8460		
Low	11(28.21)	28(71.79)				12(52.17)	11(47.83)			
High	10(31.25)	22(68.75)				17(54.84)	14(45.16)			

Abbreviations: *OR* Odds ratio, *CI* Confidence interval, *pCR* pathologic complete response, *non-pCR* non-pathologic complete response

A/EC-THP: *A/E* Anthracycline, *C* Cyclophosphamide, *T* Paclitaxel drugs, *H* Trastuzumab, *P* Pertuzumab

TCbHP: *T* Paclitaxel drugs, *Cb* platinum drugs, *H* Trastuzumab, *P* Pertuzumab

^a^Logistic regression was used for analysis. All statistical tests were two-sided. The OR was calculated using the non-pCR as a reference. Adjusted for the clinical stage, FTG_process, FTG_change

^b,c,d,e,f,g^ ROC curves refer to Fig. 2

### The subgroup analysis based on the menstrual status

The menopausal subgroup analysis showed that the clinical stage (pCR: 73.33% vs. 52.38%, OR = 0.152, 95% CI: 0.041–0.559, *P* = 0.0046), HR expression status (pCR: 77.78% vs. 53.33%, OR = 0.179, 95% CI: 0.047–0.675, *P* = 0.0111), and NAT regimen (pCR: 47.62% vs. 75.56%, OR = 4.233, 95%CI: 1.193–15.017, *P* = 0.0255) independently influenced pCR. No independent pCR predictors were present in the non-menopausal subgroup (Table 5).

**Table 5:** Analysis of the effect of the patient characteristics on pCR (Non-menopause/Menopause)

Variable	Non-menopause (*n* = 59)					Menopause (*n* = 66)				
	non-pCR N(%)	pCR N(%)	Univariate analysis *P*-value	Multivariate analysis		non-pCR N(%)	pCR N(%)	Univariate analysis *P*-value	Multivariate analysis^a^	
				OR(95%CI)	*P*-value				OR(95%CI)	*P*-value
**Total (N)**	28(47.46)	31(52.54)				22(33.33)	44(66.67)			
**Age**			0.7013					0.4868		
≤ 35	8(57.14)	6(42.86)				0	0			
35 < age ≤ 55	19(44.1)	24(55.81)				12(37.50)	20(62.50)			
> 55	1(50.00)	1(50.00)				10(29.41)	24(70.59)			
**Clinical stage**			0.5992					**0.0065**		**0.0046**
II stage	19(50.00)	19(50.00)				12(26.67)	33(73.33)		**Ref**	
III stage	9(42.86)	12(57.14)				10(47.62)	11(52.38)		**0.152** **(0.041–0.559)**	
**HR status**			0.5992					**0.0392**		**0.0111**
Negative	9(42.86)	12(57.14)				8(22.22)	28(77.78)		**Ref**	
Positive	19(50.00)	19(50.00)				14(46.67)	16(53.33)		**0.179** **(0.047–0.675)**	
**HER2 expression**			0.4010					**0.0174**		0.2347
2 + /FISH +	7(58.33)	5(41.67)				6(75.00)	2(25.00)		Ref	
3 +	21(44.68)	26(55.32)				16(27.59)	42(72.51)		3.978 (0.542–29.223)	
**Neoadjuvant therapy**			0.2215					**0.0283**		**0.0255**
A/EC-THP	10(38.46)	16(61.54)				11(52.38)	10(47.62)		**Ref**	
TCbHP	18(54.55)	15(45.45)				11(24.44)	34(75.56)		**4.233** **(1.193–15.017)**	
**FBG_baseline**^**b**^			0.5921					0.7900		
Low	9(52.94)	8(47.06)				3(37.50)	5(62.50)			
High	19(45.24)	23(54.76)				19(32.76)	39(67.24)			
**FTG_baseline**^**c**^			0.2283					0.5878		
Low	27(50.00)	27(50.00)				7(29.17)	17(70.83)			
High	1(20.00)	4(80.00)				15(35.71)	27(64.29)			
**FTC_baseline**^**d**^			0.6909					0.8155		
Low	13(44.83)	16(55.17)				4(36.36)	7(63.64)			
High	15(50.00)	15(50.00)				18(32.73)	37(67.27)			
**FBG_process**^**e**^			0.6962					0.5958		
Low	7(46.67)	8(53.33)				8(29.63)	19(70.37)			
High	21(47.73)	23(52.27)				14(35.90)	25(64.10)			
**FTG_process**^**f**^			0.9430					0.7218		
Low	16(47.06)	18(52.94)				8(30.77)	18(69.23)			
High	12(48.00)	13(52.00)				14(35.00)	26(65.00)			
**FTC_process**^**g**^			0.5466					0.6910		
Low	25(49.02)	26(50.98)				5(29.41)	12(70.59)			
High	3(37.50)	5(62.50)				17(34.69)	32(65.31)			
**FBG_change**			0.6249					1.0000		
Low	10(43.48)	13(56.52)				7(33.33)	14(66.67)			
High	18(50.00)	18(50.00)				15(33.33)	30(66.67)			
**FTG_change**			0.3175					0.5758		
Low	5(35.71)	9(64.29)				6(28.57)	15(71.43)			
High	23(51.11)	22(48.89)				16(35.56)	29(64.44)			
**FTC_change**			0.7859					0.8535		
Low	8(44.44)	10(55.68)				15(34.09)	29(65.91)			
High	20(48.78)	21(51.22)				7(31.82)	15(68.18)			

Abbreviations: *OR*, Odds ratio, *CI* Confidence interval, *pCR* Pathologic complete response, *non-pCR* non-pathologic complete response

A/EC-THP: *A/E* Anthracycline, *C* Cyclophosphamide, *T* Paclitaxel drugs, *H* Trastuzumab, *P* Pertuzumab

*TCbHP*: *T*, paclitaxel drugs, *Cb* Platinum drugs, *H* Trastuzumab, *P* Pertuzumab

^a^Logistic regression was used for analysis. All statistical tests were two-sided. The OR was calculated using the non-pCR as a reference. Adjusted for the clinical stage, HR status, HER2 expression, and neoadjuvant therapy

^b,c,d,e,f,g^ ROC curves,refer to Fig. 2

## Discussion

### FBG

Both the overall and subgroup (Wilcoxon's rank-sum test) analyses showed that although combining NAC with dual-targeted therapy increased the FBG levels (all *p* < 0.05), the mean values of the elevated levels did not exceed the normal reference ranges (normal range of FBG in the hospital: 3.89–6.11 mmol/L). The factors responsible for increasing the mean blood glucose level may be related to chemotherapeutic drug toxicity and dexamethasone pretreatment. The platinum, paclitaxel, and cyclophosphamide used during the NAT in this study kill tumor cells and damage pancreatic islet β-cells, decreasing insulin secretion, abnormal glucose tolerance, and elevated blood glucose. In addition, since patients receiving glucose-lowering therapy were not excluded from this study, it was hypothesized that the effect of NAT on FBG might be even more significant. Therefore, BC patients receiving combined NAC and dual-targeted therapy should pay close attention to their blood glucose levels. Further association analyses of the overall and multiple subgroups showed no association between FBG and pCR (*p* > 0.05), indicating that NAT increased the FBG levels, while FBG could not predict pCR.

### FTG and FTC

#### FTG

Both the overall and subgroup (Wilcoxon's rank-sum test) analyses showed that combined NAC and dual-targeted therapy increased the FTG levels (all *p* < 0.05). Except for the PR-positive and non-menopausal subgroups, the mean FTG level of the overall and other subgroups exceeded the reference range (normal range of FTG in our hospital: 0.00–1.70 mmol/L) after NAT. The logistic regression analyses indicated that low levels of FTG during NAT and decreased FTG after NAT predicted higher pCR rates in ER-negative/HER2-positive and PR-negative/HER2-positive patients, respectively (*p* < 0.05). In addition, since patients who received triglyceride-lowering therapy were not excluded from this study, the FTG levels of all patients exposed to combined NAC and dual-targeted therapy should be closely monitored. They should receive possible triglyceride-lowering therapy in a timely manner, especially ER-negative/HER2-positive and PR-negative/HER2-positive patients with BC, since their triglyceride-lowering therapy possibly enhances pCR.

#### FTC

The overall analysis (Wilcoxon's rank-sum test) showed that combined NAC and dual-targeted therapy decreased the FTC levels. In addition, although the FTC levels increased in the ER/PR-positive subgroups and decreased in the ER/PR-negative subgroups, the differences were not statistically significant (all *p* > 0.05). The subgroup analysis of the menstrual status showed that combined NAC and dual-targeted therapy decreased the FTC levels in the menopausal group while increasing those in the non-menopausal group (both *p* < 0.05) (normal range of FTC in the hospital: 0.00–5.20 mmol/L). Further, overall and multi-subgroup logistic regression analyses indicated that FTC did not independently influence pCR (*p* > 0.05). Therefore, although NAT elevated the FTC levels, FTC did not predict pCR.

### Summaries

In terms of NAT affecting lipids, previous studies have shown that (neo)adjuvant therapy leads to elevated FTG and FTC levels in BC patients [23–27]. However, our results were only partially the same as previous studies, as we found that FTG was significantly elevated in both overall and subgroups (both *p* < 0.05), but FTC was significantly elevated only in the non-menopausal subgroup (*p* < 0.05) and decreased in the menopausal group (*p* < 0.05). The possible reasons for the differences were analyzed as differences in treatment regimens, molecular typing and enrollment criteria. In terms of lipid prediction of pCR, our study found that FTG in process and change groups could only predict pCR in some HER2-positive BCs. On the contrary, unlike our study, some studies [23] found no significant association between lipid levels and pCR rate in both baseline and process groups, which may be related to different group stratification methods and study backgrounds. Since, as far as is known, this is the first high-quality retrospective study on FTG and FTC based exclusively on combined NAC and dual-targeted therapy in the context of HER2-positive BC, there is a lack of reference to previous studies.

It should be noted that lipid is a general term for serum FTG, FTC, and lipoid, and FTG and FTC, as the most clinically relevant lipids, are not only associated with the development of CVD, but also with a multiple of developmental processes such as cell growth, proliferation, differentiation, apoptosis, motility, and metastasis in BC [28–31]. In HER2-positive breast cancer, fatty acid accumulation due to dyslipidemia also promotes drug resistance via acyl-CoAcholesterolacyltransferase and apolipoprotein E receptors [32]. In addition, CVD is an important cause of death after a diagnosis of BC, and women with BC have a higher risk of developing CVD than women in the general population [33, 34]. Therefore, controlling FTG and FTC at reasonable levels is crucial for BC patients treated with NAT.

### Clinical and pathologic

In addition to biochemical indexes (FTG), this study indicated that the clinical stage, treatment regimen, and expression status of HER2 and HR (all *p* < 0.05) displayed good pCR predictive in some cases, which was consistent with previous studies.

### Limitations

Since this was a single-center retrospective study, the case samples were small, and bias might be evident in case selection, possibly influencing the results. In addition, limited by the lack of high-density lipoprotein, low-density lipoprotein, and very-low-density lipoprotein information, further subgroup analysis could not be performed. Finally, this study was more concerned with describing the statistical differences and did not explore the molecular mechanisms underlying the association between biochemical indicators and pCR. Therefore, further multicenter prospective studies with high-quality, large samples and basic research are necessary.

## Conclusions

Combined NAC and dual-targeted therapy increase the overall and subgroup FBG and FTG levels (*p* < 0.05), while the FTC was lower in the menopausal group and higher in the non-menopausal group (*p* < 0.05). In addition to the clinicopathologic features, the lower FTG level during and after treatment could be used as an independent influence to predict ER-negative and PR-negative pCR, respectively (*p* < 0.05). Overall and subgroup analyses showed that FBG and FTC did not predict pCR (*p* > 0.05). Early and timely intervention of FTG levels based on pathologic features may improve the pCR rate and reduce the likelihood of long-term CVD risk in HER2-positive BC treated via combined NAC and dual-target therapy.

## Data Availability

No datasets were generated or analysed during the current study.

## Abbreviations

A/E: Anthracycline
AUC: Area under curve
BC: Breast cancer
C: Cyclophosphamide
Cb: Platinum drugs
CI: Confidence interval
CVD: Cardiovascular disease
EFS: Event-free survival
ER: Estrogen receptor
FBG: Fasting blood glucose
FISH: Fluorescence in-situ hybridization
FTC: Fasting total cholesterol
FTG: Fasting triglyceride
H: Trastuzumab
HER2: Human epidermal growth factor receptor 2
HR: Hormone receptor
IHC: Immunohistochemistry
NAC: Neoadjuvant chemotherapy
NAT: Neoadjuvant therapy
non-pCR: Non-pathologic complete response
OR: Odds ratio
OS: Overall survival
pCR: Pathologic complete response
P: Pertuzumab
ROC: Receiver operating characteristic
RP: Progesterone receptor
SAS: Statistical analysis
T: Paclitaxel drugs
